# The truth about 17-beta estradiol: menopause beyond “old wives’ tales”

**DOI:** 10.3389/fendo.2023.1229804

**Published:** 2023-09-11

**Authors:** Lawrence M. Nelson

**Affiliations:** Digital Women's Health Initiative, Mary Elizabeth Conover Foundation, Inc., Tysons, VA, United States

**Keywords:** 17 beta-estradiol (E2), women’s health, public health, policy and administrative challenges, physiologic primary ovarian insufficiency, menopausal hormonal therapy, morbidity & mortality, FDA boxed warning

## Introduction

An “old wives’ tale” is a traditional truth not based on fact (1). The term is now an outmoded and disrespectful vernacular about women (2). Many spurious truths about menopause have become culturally embedded. To improve understanding and provide better care, a new perspective, and more accurate biologic terminology is needed for “*the menopause*.”

A recent report in *The New York Times*, “Women have been misled about menopause,” (3) demonstrates this subject remains a difficult conversation (4, 5). Importantly, women younger than 45 who experience signs and symptoms of menopause have a metabolic derangement associated with increased morbidity and mortality (6, 7). The typical midlife hormonal transition women experience, in many cases, adversely impacts their quality of life and creates economic burdens (8, 9).

The truth usually lives between arbitrary categories (10). The categorical and absolute terms “premenopausal” and “postmenopausal” are problematic, misleading, and sources of confusion. The midlife physiologic hormonal transition women experience is a complex process, gradually evolving and constantly in flux. This transition takes years to develop and is not two static states, one before and one after the last menses. Categorical thinking treats the categorical structure imposed as if the categories are static states (11). This way of thinking prevents clear communication and effective management. Another unhelpful way of categorical thinking is viewing the midlife physiologic hormonal transition as purely dichotomous, as either 1) a natural event needing no medical intervention or 2) a disease needing active intervention (11).

The social experience of the midlife transition in women related to the cessation of menses is culturally mediated (12). Menopause is a fascinating biological phenomenon that has puzzled scientists for decades. Women live many years after their reproductive capacity has ended (13). Researchers still debate the nature of the evolutionary pressure that caused menopause to develop in women. Essentially, this midlife transition has adaptive advantages by redirecting resources away from the dangers of reproduction at a later age and toward other essential and less risky needs of family and society, such as being a source of wisdom (13–15). Regardless of the exact reasons, post-reproductive longevity highlights the complexity of aging and the importance of understanding this phenomenon in greater depth. This midlife physiologic transition is an integral part of a woman’s life and has played a vital role in the survival and evolution of our species.

The essential biology of this physiologic midlife transition in women is a waning of ovarian endocrine function. The biological basis for the timing of this normal transition is the decline and eventual exhaustion of the number of potentially functional ovarian primordial follicles (16). The essential endocrinology involved is transitioning away from high average serum levels of 17-beta estradiol (E2) during reproductive years. These high levels support fertility and maintain health and resilience when menses are regular and ovulatory. The midlife physiologic transition is toward dramatically lower E2 levels in the later years, levels which are known to contribute to increased health risks. While menopause is the permanent cessation of menses and reproductive capacity, there is much more to it concerning public health (17).

Accumulating evidence begs an essential public health question. “Is there a safe and effective selection process and treatment regimen to spare those women at risk of the detrimental effects of low E2 in later life?” Evidence is clear, extremely low E2 levels increase the risk for some women. For example, there is a 2.5-fold increase in hip and vertebral fractures in older women with total E2 levels less than 5.0 pg/ml. Men have a similar association between E2 levels and fractures (18). Intriguingly, even minimal increases in E2 serum concentrations have a proven beneficial effect on bone mineral density in menopausal women, with little effect on endometrial proliferation (19, 20). These observations suggest a potentially safe therapeutic window of low physiologic E2 replacement may exist in other areas of a woman’s health. This same therapeutic window of low doses of E2, proven to improve bone mineral density, could theoretically also improve health for menopausal women regarding their cardiovascular health, central nervous system health, mood, and related cosmetic benefits to skin and hair (21, 22).

Accumulating evidence begs another essential public health question. “Is there a unifying biologic terminology to describe the physiology of ovarian hormone function across a woman’s lifespan?” The answer is clearly yes. More than 80 years ago, Fuller Albright, an endocrinologist at Harvard University, provided us with the scientific language for this purpose (23). He used the term “*Primary Ovarian Insufficiency*” to first define the scientific mechanism of failure of pubertal development in Turner Syndrome. His work showed that the pathologic defect was in the ovaries, not the hypothalamus or pituitary. Some women experience normal puberty and then develop amenorrhea due to *Primary Ovarian Insufficiency* before age 40 (7). In scientific terms, women in the midlife physiologic hormone transition are experiencing “*Physiologic Primary Ovarian Insufficiency.*” This midlife transition is a process, not an event.

## Transitioning to a scientific perspective

P-POI (Physiologic POI) is a transition away from reproduction risks. Menopause is a categorical, absolute state. P-POI is a transition providing women the freedom to undertake other contributions to family and society based on accumulated wisdom. Speaking truth charts a path to being well. As noted, there is confusion and misinformation about the role of estrogen in women’s health and, specifically, the management of E2 deficiency in women across the lifespan (3, 5). Notably, the scientific community recognizes many biological effects of the most potent endogenous estrogenic sex steroid hormone, E2 (24). These hormonal effects go beyond reproduction and carry broad and profound public health implications.

A hallmark of science is the ability to self-correct. Deviation from seeking the truth as the priority seriously impairs the ability of science to self-correct (25). This self-correction may take decades in some cases and requires new evidence and new ways of thinking. The process does not happen by default. Emphasizing the political nature of progress, another example of categorical thinking impairs progress in social justice and health (26). Correcting misunderstandings regarding the effects of E2 deficiency on public health requires clear insight into the best evidence about E2 and creating more and better evidence in the future. Getting this evidence will require a clear and unifying global strategy for women’s health (27).

The physiologic midlife transition to menopause is a state of low serum E2, and early menopause is associated with significant morbidity and early mortality (28, 29). A few prospective population-based cohort studies provide convincing evidence that women with early onset menopause, and the associated E2 deficiency, have 1) a shorter life expectancy, 2) increased risk of type II diabetes, 3) adverse effects on cognitive function, 4) significant correlation between age at menopause and age at diagnosis of dementia, and 5) a significant correlation between age at menopause and age at death (6, 30, 31). This midlife transition to ultra-low serum E2 levels is associated with significant cardiovascular morbidity and mortality. Prospective epidemiologic studies correlate earlier menopause with earlier death, ischemic stroke, and a combined effect of earlier menopause and high-risk factors on death and cardiovascular disease, osteoporosis, and fragility fracture (32–34).

### Physiologic Primary Ovarian Insufficiency:

#### A continuum of women’s health across the lifespan

As noted above, in 1942, Fuller Albright used the term “*Primary Ovarian Insufficiency*” when he first reported a condition involving cessation of normal ovarian function in young women (23). He concluded that the defect in function in these patients was primarily in the ovaries when his bioassay revealed high urinary FSH levels. Endocrinologists generally classify disorders of endocrine glands as “primary” when the *defect within the gland renders the gland unresponsive to stimulation. On the other hand, the central component of the axis (hypothalamus and pituitary) functions normally.*

Hence, in *Primary Ovarian Insufficiency* (POI), serum gonadotropin levels are high due to the absence of feedback inhibition from the ovary. This same biology is the situation in women undergoing the age-related physiologic transition to waning ovarian function. From this perspective, the midlife transition to the cessation of menses in women is a natural and physiologic process of “*Physiologic Primary Ovarian Insufficiency*” (P-POI), programmed by the decline and exhaustion of ovarian follicle number (**Table 1**).

**Table 1:** The Physiology and Pathophysiology.

Primary Ovarian Insufficiency (POI) Physiologic POI (P-POI) - Onset after age 40							
Physiologic State	Menstrual Cycle	Age Years	AMH^a^ ng/ml		AFC^b,c^	FSH^d,e^ IU/L	Response to FSH Stimulation
Normal	Regular	25 to 30	2.0 to 3.0		12	5.0	Normal
Normal	Regular	30 to 35	1.5 to 2.0		11	5.0	Normal
Occult POI	Regular	35 to 40	1.0 to 1.5		9	5.0	Impaired
Biochemical POI	Regular	40 to 45	0.25 to 1.0		6	High	Impaired
PhysiologicPOI	Irregular	45 to 60	< 0.1 to 0.25		2	High	Impaired to Nil
Mature POI	Absent	> 60	< 0.1		0	> 25 to 40	Nil
Pathologic Early POI (E-POI) - Onset before age 40							
Physiologic State	Menstrual Cycle	Age Years	AMH^a^ ng/ml	AFC^b,c^		FSH^d,e^ IU/L	Response to FSH Stimulation
Pubertal Delay	Absent	13	Low	Low		High	Impaired to Nil
Primary Amenorrhea	Absent	15	Low	Low		High	Impaired to Nil
Occult POI	Regular	13 to 40	Normal	Normal		Normal	Impaired
Biochemical POI	Regular	13 to 40	Low	Low		High	Impaired
Overt POI (E-POI)	Irregular	13 to 40	Low	Low		High	Impaired

a Approximate theoretical mean ranges based on published evidence.

^b,c^Approximate theoretical 50th percentile based on published evidence.

^d,e^Approximate theoretical mean or assay-dependent threshold for diagnosis based on published evidence.

It appears serum AMH level identifies the endocrinologic leading edge in the age-related decline in ovarian function, which is best termed P-POI (35). When this process occurs before age 40, the unifying term would be Early POI (E-POI). In a longitudinal study of a cohort of women in midlife who had repeated hormone measures and experienced natural menopause, AMH decreased markedly before menopause and preceded the increase in FSH. Thus, AMH is an earlier and more sensitive marker of the POI, whether pathologic at an early age or physiologic at midlife (35).

### Pathologic Primary Ovarian Insufficiency:

#### A continuum of women’s health across the lifespan

In 1938, Henry Turner published cases of what today is known as Turner Syndrome (36). The paradigm clinical presentation is a young girl who fails to enter pubertal development, i.e., no breast development and no first menses. At the time of Turner’s report, there was debate about whether the cause was dysfunction centrally (hypothalamus/pituitary) or peripherally (ovaries). Fuller Albright settled this debate by publishing evidence of high urinary FSH levels in the syndrome. Thus, Albright documented the first Early Primary Ovarian Insufficiency (E-POI) cases (23) (**Table 1**).

I saw my first case of pathologic E-POI while in private practice in Lynchburg, Virginia, USA. The woman also had autoimmune thyroiditis, which intrigued me as a possible case of polyglandular autoimmune syndrome. This experience changed the course of my career. Eventually, my interest in the enigma brought me to the Intramural Research Program (IRP) of the US National Institutes of Health (NIH). I worked as a Principal Investigator in the NIH Clinical Center. For many years I led a multidisciplinary team to take an integrated approach blending basic science with clinical trials. The *New England Journal of Medicine* requested I write a Clinical Practice review on the condition (7). Now, after I retired from the NIH in 2017, I have been advancing the cause of E-POI as President of the Mary Elizabeth Conover Foundation, Inc (37, 38). Our mission is to establish a global digital medical hub for E-POI clinical care and natural history research, as recommended more than 10 years ago by the proceedings of an NIH conference on the subject (39).

### The basic science of 17-beta estradiol action

#### Actions of E2 in men

A recent genome-wide association study demonstrated a causal effect of endogenous serum E2 levels on increased bone mineral density in both men and women (40). Another recent genetic study showed evidence that higher serum E2 levels in men are associated with a reduced incidence of a thromboembolic phenomenon and a lower incidence of ischemic stroke (41). Surprisingly, men matched for age with menopausal women have higher serum E2 concentrations (42). In men, biological actions formerly attributed to testosterone may be actions of E2 resulting from the aromatization of testosterone (43). In this sense, testosterone is a “prehormone” in men. Furthermore, many male somatic and reproductive tissues express E2 receptors. In the rare cases of men lacking aromatase or a functional estrogen receptor alpha, evidence suggests vital actions of E2 in regulating the insulin-like growth factor-1 axis, bone growth, maintenance of skeletal health, body composition, glucose metabolism, vasomotor stability in addition to regulating male reproductive function and the hypothalamic-pituitary-testicular axis (43).

#### Actions of E2 in women

##### Actions of E2 in the brain

Evidence associates shorter cumulative life exposure to E2 in women with a higher risk of dementia (44). Notably, the scientific community now recognizes the neuromodulator effects of the potent sex steroid hormone E2 (24). E2 induces both spinogenesis and synaptogenesis (45, 46). These actions are mediated not only by classical slow-acting genomic effects by ER α and ER β receptors but also by rapid activity by membrane-bound ER α, ER β, and G protein-coupled estrogen receptor 1 (GPER1) mediating immediate nongenomic effects (47).

In female animal models, considerable evidence supports the crucial role of E2 in regulating learning and memory. A growing body of literature indicates a similar role in male animals. In animal models, E2 signaling affects spatial memory, object recognition memory, social memory, and fear memory (24). In female mice, E2 reduces anxiety by activating estrogen receptor-β (ERβ) (48). These laboratory science findings may have important clinical implications for managing E2 deficiency in women across the lifespan. In female rodents, E2 rapidly activates numerous cellular events in the brain, including cell signaling, histone modification, and local protein translation. This rapid E2 action in the brain consolidates spatial and object recognition memories (49). E2 also facilitates higher cognitive functions by exerting effects on the prefrontal cortex.

The evidence in animal models begs, “Is there a minimal neuroprotective level of E2 in humans?” The E2 effects related to higher cognitive function and synaptic health go well beyond the traditional role wrongly confined to reproduction. These findings support the critical role of later-life E2 deficiency in exacerbating the effects of aging on cognitive functions. Studies in nonhuman primate models are relevant for developing effective interventions for menopausal women (47).

##### Actions of E2 in the heart and cardiovascular system

The leading cause of death in women is cardiovascular disease, and there is a notable increase in the risk for this disease after menopause (50). Presenting myocardial infarction and stroke symptoms differ between men and women. Traditional symptoms defined in men are less commonly observed in women. For example, women are more likely than men to report nausea, vomiting, referred pain, cough, and fatigue when experiencing a myocardial infarction and less likely than men to report chest pain or sweating. These acute situations must be recognized and treated expeditiously. Delay in care leads to worse outcomes. Work remains to ensure early recognition of myocardial infarction and stroke in women (51, 52).

E2 plays a significant role in the modulation of cardiovascular physiology and pathophysiology (53). E2 regulates related gene expression, contractile function, microvascular function, metabolic processes, and calcium signaling (53). Notably, a prospective controlled study providing transdermal physiologic E2 replacement to menopausal women (in a manner mimicking premenopausal levels) significantly lowered blood pressure (54). The G protein-coupled estrogen receptor 1 (GPER1), expressed ubiquitously in the cardiovascular system and activated by E2, mediates vascular, renal, and cardiac mechanisms that influence blood pressure regulation and are active in vasodilation (55).

The evidence begs the question, “Is there a minimal cardiovascular protective level of E2 in humans?” Investigators in the SWAN Heart Study (Study of Women Across the Nation) detected changes in arterial stiffness within one year of the final menstrual period (56). The Multi-Ethnic Study of Atherosclerosis (MESA), a prospective cohort study, used Cox hazard models to evaluate associations of E2 with the outcome. After adjusting for demographics, risk factors, and use of hormone therapy, they found higher E2 levels associated with a lower risk of coronary artery disease (57). A longitudinal study of midlife women over up to 9 years showed that those with lower endogenous E2 levels had increased subclinical atherosclerosis progression (58). Vasomotor symptoms (hot flashes and night sweats) related to E2 deficiency are an independent indicator of increased risk of coronary artery disease after correcting for traditional cardiovascular risk factors (59).

##### Actions of E2 in bone

E2 is the crucial regulator of bone metabolism in both men and women. Bone, a metabolically active and complex tissue, plays five significant roles in health:

1. Mechanical, i.e., support and locomotion
2. Protection of vital organs (brain, heart, lungs)
3. Hematopoietic
4. Metabolic, as a reservoir for calcium, phosphorus, and mineral ions
5. Endocrine, i.e., the production of osteocalcin and FGF23 (60, 61)

Osteoclasts and osteoblasts are highly differentiated cells. Osteoclasts resorb bone; osteoblasts produce new bone. Osteoblasts transition to osteocytes, mechanosensory cells able to sense and respond to mechanical forces (62). Bone modeling, a life-long process, involves cycles of bone resorption and formation and determines bone health, development, and maintenance. Maintenance of bone mass throughout life requires a strict balance between bone resorption and formation linked in time and space. This strict correlation between bone resorption and formation is called coupling, with an adequate number of osteoblasts forming at resorption sites (63). Peak bone mass, an E2-dependent process, is an essential predictor for bone strength and osteoporotic fracture risk later in life. Peak bone mass is acquired at the end of the adolescent growth period (64).

In adolescent girls and women, an abrupt decline in serum E2 levels is associated closely with increased osteoclastic bone resorption. Low E2 levels stimulate circulating macrophages to produce osteoclastic cytokines that activate RANK and promote osteoclast activation. Additionally, the loss of direct pro-apoptotic effects of E2 on osteoclasts prolongs osteoclast lifespan, accelerating trabecular bone loss (65, 66).

The midlife physiologic transition to P-POI and the accompanying loss of E2 are associated with bone mineral density declines. E2-dependent bone loss has a predictable pattern. Initially, the loss is relatively rapid, affecting trabecular bone preferentially. After 10 to 15 years of E2 deficiency, the skeletal mass may be one-third to one-half of peak bone mass. The resulting skeletal fragility permits even minimal trauma to cause spine and wrist fractures. In later years hip fractures are alarmingly frequent (67). Timely restoration of E2 levels prevents E2-dependent bone loss and significantly reduces fracture risk. High calcium intake in the face of deficient E2 is ineffective in reducing the risk of bone fracture across the menopause transition (68).

While little recognized and little employed, published evidence demonstrated many years ago that there is a very low dose of E2 (only 14 micrograms per day) administered by a transdermal patch that effectively protects bone density in menopausal women (19, 20). Long-term studies confirm that women in midlife who regularly use menopausal hormone therapy have greater bone mass and fewer osteoporotic fractures (69). A recent meta-analysis clarified the effects of transdermal E2 replacement on BMD in menopausal women. According to pooled estimates, transdermal E2 significantly increased lumbar spine BMD one and two years after initiation of therapy (70).

### Physiologic hormone replacement

Physiologic replacement of 17-Beta Estradiol is administered transdermally or transvaginally to avoid adverse “hepatic first-pass effects” of oral administration of estrogens (71). Oral estrogens are known to increase the risk of potentially fatal thromboembolic events (71). In 1986, Judd and colleagues published evidence indicating that transdermal estradiol can elicit many of the desirable actions of estrogen while avoiding the pharmacologic effects of oral estrogens on hepatic proteins (72). For this reason, when the NIH POI research team initiated clinical studies of pathologic POI in 1991, they chose the transdermal route of estradiol administration as the safer alternative. Prospective, randomized, double-blind, controlled studies provide the best evidence for clinical decisions (73). Over the past 20 years, the US National Institutes of Health (NIH) Intramural Research Program (NIH-IRP) conducted the only such study on hormone replacement in women with pathologic early POI (74). The study provided these women with the average daily production rate of estradiol (100 micrograms per day) by transdermal patch and cyclic, monthly oral progestogen. Over the three-year study, the NIH-IRP hormone replacement regimen restored bone mineral density to normal. Women tolerated the treatment well.

Physiologic E2 replacement is also available for women in the midlife transition, i.e., what we are terming, Physiologic Primary Ovarian insufficiency (P-POI). Physiologic E2 replacement is proven safe and effective for women experiencing the midlife transition symptoms of E2 deficiency, such as vasomotor instability and vaginal atrophy (75–77). Physiologic E2 in the low dose administered by the patch is also proven safe and effective in mitigating midlife transition bone loss as prevention of osteoporosis (19, 20).

### A change in course: the US Women’s Health Initiative

Women’s health issues other than reproduction received little attention and little research funding before 1986. These omissions are regrettable regarding heart disease, long considered less risky for women than men (78). Coronary artery disease (CAD) is the most common reason for death in men and women in the US. Trends in the US suggest one-half of 40-year-old men will develop CAD in the future, and one in three healthy 40-year-old women (79).

In a 1990 report in the journal *Science*, this “malign neglect” of women’s health was attributed to the NIH (80). The awareness shifted focus toward women’s health issues and a greater interest in understanding how heart disease might affect women differently than men. The NIH effort seemed to some to be “too little too late” to address the issue of women’s participation in research. In 1991, Dr. Bernadine Healy, newly appointed as the first woman director of the NIH, announced her plan for the *Women’s Health Initiative* (WHI). She obtained funding directly from Congress as a discrete line item, with a projected budget of $625 million over the life of the 15-year study (81).

The initiative set out to bring greater attention to the significant causes of death and disability among women, particularly those related to heart disease, cancer, and osteoporosis. The funding by Congress provided the necessary resources for establishing several clinical trials involving thousands of women across the United States. The WHI has increased attention to the pressing need to address the unique health concerns of women. Indeed, the United States Government established the US *Office of Women’s Health* (OWH) as part of the Department of Health and Human Services. Its mission is to improve the health of women and girls through advocacy, education, and research (82).

In this role, the OWH has played a critical role in advancing women’s health issues and addressing health disparities that affect women. Over the years, the office has launched numerous initiatives to improve women’s health outcomes, including campaigns to increase awareness about breast cancer, heart disease, and other health issues that disproportionately affect women. The OWH has also been instrumental in advocating for policies and programs that promote women’s health, such as the Affordable Care Act, which expanded access to preventive services like mammograms and contraception. Today, the OWH remains a crucial player in the fight for women’s health equity and remains committed to improving the health of all women and girls across the United States.

The WHI also aimed to expand research on the significant causes of death, disability, and frailty among women. Thus, Congress also established the *Office of Research on Women’s Health* (ORWH) as part of the US *National Institutes of Health* (NIH) (83). Congress recognized that women’s health research needs more funding and more inclusion of women in clinical trials. These deficiencies created a gap in knowledge about women’s health. The ORWH promotes and supports research on women’s health and differences based on sex and gender. It works to ensure that all NIH-funded research considers sex as a biological variable and advocates for women’s inclusion in clinical studies. The ORWH also funds research on conditions that disproportionately affect women, such as breast cancer, osteoporosis, and autoimmune diseases. Through its efforts, the ORWH has made significant contributions to advancing women’s health research and improving the health outcomes of women.

### Critical appraisal: the US NIH Women's Health Initiative

Critics give mixed reviews about the WHI. Some praise the efforts to improve women’s health. In contrast, others have criticized its origins as based more on political correctness and less on good scientific methods, and some have seriously questioned the validity of its results (84). This 1993 report in the prestigious journal Nature went so far as “Critics condemn NIH women’s study.” (84) The headline read, “NIH may be wasting $625 million or more in pursuit of an ambition to be seen to be politically correct.” This severe criticism of the NIH WHI came from a committee of the Institute of Medicine appointed by Congress to appraise one of NIH’s most expensive programs ever. The report was *“a scathing indictment of its conception and design.*” The committee criticized the WHI on several fronts based on (I) scientifically weak premises (2); flawed statistically by poor design (3); inadequate informed consent; and (4) a probable underestimate of the true cost. Surprisingly, the committee acknowledged politics rather than science and had the committee come short of an outright call to cancel the NIH WHI study.

A major concern with the design of the NIH WHI study was the age of the participants. In the WHI studies, women averaged approximately 12 years after menopause. Many of these women would have had significant asymptomatic atherosclerosis upon entry into the trial. Substantial data demonstrate athero-preventive effects of estrogen before vascular damage occurs, whereas adverse effects of oral estrogen on thrombosis and inflammation may predominate once complex atheromas are present. As a result, the study was not a primary prevention trial. From this perspective, the study may have caused great harm to participants (85).

The NIH WHI study has significantly impacted women’s health, but many unanswered questions and controversies remain. Not all the impact has been salutary to women’s health. Despite criticisms, the US WHI remains an essential milestone in women’s health history, some for good, some otherwise.

When it comes to conducting clinical research, ethics play a crucial role. Flawed research can have serious consequences, from misleading patients to wasting valuable resources. That is why ensuring that all research is conducted ethically is so important. One key ethical concern is the validity of the research itself. The results may be inaccurate or unreliable if a study is flawed. This can have serious implications for patients and the wider medical community. Researchers must take great care to design studies that are scientifically sound and free from bias. Another important ethical issue is patient safety. Clinical research often involves testing new treatments or procedures on human subjects. It is essential that patients are fully informed of the risks and benefits of participating in the research and that their safety is always a top priority.

Flawed clinical research with inadequate informed consent is unethical research. The quality of the informed consent obtained from the WHI participants is of particular concern. Were these women informed regarding the availability of safer transdermal E2 alternatives to the oral estrogen used in the study? There has been a call for an independent investigation of the NIH WHI to scrutinize every major WHI paper to determine whether the data justified the conclusions drawn (86). It may be time to call on Congress to initiate the process.

## FDA black box warnings on estrogen

### A problematic legacy of the US NIH Women’s Health Initiative

These FDA black box warnings on E2 may cause significant problems for patients and healthcare providers. Some see these warnings as a blatant interference with the mutual respect and trust between the individual patient and her clinician. Two glaring examples of what may be termed “heavy-handed regulation” are situations of administration of very low doses of E2 administered physiologically and with proven clinical benefit outweighing the risk. The first example is using 0.014 micrograms of transdermal E2 to prevent bone loss, an FDA-approved treatment (19, 20). What is the necessity for a boxed warning on such a low physiologic dose? The FDA black box warning on this minimal dose of E2 may have put millions of women at unnecessary risk of osteoporosis. A second glaring example is using the vaginal E2 ring for urogenital atrophy, which has proven low systemic absorption (87). What is the need for a boxed warning in this case? These warnings may likely cause many women to suffer needlessly in fear of proven treatment.

There are many avenues of concern regarding these box warnings on E2. First, the alarm may discourage providers from prescribing the medication, even if it is the most appropriate treatment for their patient. Second, the warning may cause patients to fear taking the therapy, leading them never to start the treatment or to stop the treatment altogether without consulting their clinician. Such action can be dangerous for treatments critical to their health. Third, the black box warning may discourage pharmaceutical companies from producing or marketing treatments of proven benefit.

FDA warnings may even have had broader public health spillover effects. For example, the National Institutes of Health Women’s Health Initiative Study and the subsequent FDA box warnings on estrogen caused menopausal hormone use to drop sharply. One analysis showed associated spillover effects of the WHI study on preventive care visits by women aged 60–69, who had statistically significant declines in their mammography, cholesterol, and blood stool tests (88).

Overall, issuing a black box warning is a serious matter that requires careful consideration of the potential benefits and risks of the treatment. Of concern, no uniform FDA guidelines exist on removing boxed warnings. To promote the safe use of approved therapies, the FDA should adopt a uniform and transparent process governing decisions to impose or, importantly, to remove boxed warnings when they cause more harm than good (89).

Paradoxically, the FDA black box warnings divert women away from FDA-approved hormone replacement. Instead, fear makes women vulnerable to unregulated therapies with unproven benefits and unknown risks as provided by purveyors of “Compounded Bioidentical Hormone Therapy.” (90)

## Conclusion

*“Physiologic Primary Ovarian Insufficiency”* is a unifying scientific term describing the physiology and trajectory of ovarian hormone function across a woman’s lifespan into her later years. To improve understanding and provide better care, a new perspective and this more accurate biologic terminology is needed for “*menopause*.” The essential biology of this physiologic midlife transition in women is a waning of ovarian endocrine function. The physiologic midlife transition to so-called *menopause* is a state of low serum E2, and early E2 deficiency is associated with significant morbidity and early mortality. A change in terminology away from the culturally defined *menopause* and to the scientifically defined *Physiologic Primary Ovarian Insufficiency* (P-POI) is needed to increase awareness of the underlying biology and endocrinology, improve communication, and provide more effective evaluation and management of the associated role of physiologic E2 replacement in maintaining a woman’s health.

## Author contributions

The author confirms being the sole contributor of this work and has approved it for publication.
